# Children’s functional performance barefoot and in sports shoes

**DOI:** 10.1186/1757-1146-5-S1-P31

**Published:** 2012-04-10

**Authors:** Caleb Wegener, Andrew Greene, Renee Millar, Joshua Burns, Adrienne E Hunt, Benedicte Vanwanseele, Richard M Smith

**Affiliations:** 1 Discipline of Exercise and Sports Science, Faculty of Health Sciences, The University of Sydney, NSW, 1825, Australia; 2 Faculty of Health Sciences, The University of Sydney/ Institute for Neuroscience and Muscle Research, The Children’s Hospital at Westmead, Sydney, NSW, 2145, Australia; 3 Research Centre for Exercise and Health, KULeuven, Leuven, Belgium / Chair Health Innovation and Technology, Fontys University of Applied Sciences, Eindhoven, Netherlands

## Background

Shoes have a considerable impact on children’s walking and running biomechanics [1]. Given the number of significant changes shoes make to children’s gait, shoes may also affect children’s ability to perform functional tasks. This study aimed to determine the effect of shoes on children’s balance, standing long jump and running agility.

## Methods

Nine boys and 10 girls (mean age 10 years (SD1.4)) performed four activities barefoot and wearing sports shoes (Kanbarra, Asics Oceania Pty Ltd.) in a randomised order. To allow for a gradual warm up, activities were undertaken in a predetermined order of: 2x20sec single leg balance eyes open; 2 x20sec single leg balance eyes closed; 2x standing long jump; timed running agility (4x10m). These tasks were undertaken as previously described in the literature [2,3]. The balance tasks were undertaken on concrete surface, while the standing long jump and agility were undertaken on carpet. The better of two performances, except running agility in which only one attempt was undertaken, were selected for analysis. Significance was assessed with the Wilcoxon Signed rank test for non-parametric data and a paired samples t-test for parametric data.

## Results

Shoes did not significantly alter single leg balance with eyes open (20sec (0) to 20sec (0); *p*=1.00) or with eyes closed (12.4sec (7.9) to 14.4sec (7.4); *p*=0.255). Children jumped further in shoes (1.43m (0.24) to 1.48m (0.23); *p*=0.033). Running agility did not significantly change in shoes (12.8sec (1.1) to 12.9sec (1.4); p=0.250).

## Conclusions

Shoes improve children’s standing long jump performance. This is possibly due to increased perception of protection on landing or improved friction between the outersole and carpet. Alternatively the splinting effect of shoes could improve force transfer from the calf musculature to the foot and ground. Sports shoes do not impair static balance or agility but do improve children’s standing long jump.
